# Transcriptome Analysis of Thiram-Treated Zebrafish (*Danio rerio*) Embryos Reveals Disruption of Reproduction Signaling Pathways

**DOI:** 10.3390/biology12020156

**Published:** 2023-01-19

**Authors:** Bala Murali Krishna Vasamsetti, Kyongmi Chon, Ji-Yeong Choi, Juyeong Kim, Chang-Young Yoon

**Affiliations:** Toxicity and Risk Assessment Division, Department of Agro-Food Safety and Crop Protection, National Institute of Agricultural Sciences, Rural Development Administration, Wanju-gun 55365, Republic of Korea

**Keywords:** developmental toxicity, pesticide toxicity, reproductive toxicity, thiram, transcriptome analysis, zebrafish

## Abstract

**Simple Summary:**

Thiram is a widely used fungicide, yet studies have shown that it has hazardous effects on animals, including fish. We performed developmental and transcriptome analyses of thiram-exposed zebrafish embryos to further understand thiram toxicity. Thiram-exposed embryos exhibited several developmental abnormalities. Transcriptome analysis revealed 1754 differentially expressed genes associated with thiram exposure when compared to controls. Kyoto Encyclopedia of Genes and Genomes pathway enrichment analysis of thiram-treated samples highlighted reproduction-related pathways. The current findings shed light on the molecular mechanisms through which thiram exerts developmental and reproductive toxicity in zebrafish.

**Abstract:**

Thiram, a dithiocarbamate fungicide, is used for the treatment of various fungal infections affecting crops and ornamentals. However, thiram-associated toxicity has been reported in animals, including fish, and the underlying molecular mechanisms are unclear. Herein, we employed zebrafish (ZF) to gain further insights into thiram toxicity-associated molecular mechanisms. We studied developmental abnormalities and performed whole-transcriptome analysis of ZF embryos exposed to thiram for 96 h. Embryos exposed to 4.0 μg/L thiram exhibited several phenotypic abnormalities, including bradycardia, spinal curvature, hatching arrest, and growth retardation. Whole-transcriptome analysis revealed 1754 differentially expressed genes (DEGs), with 512 upregulated and 1242 downregulated DEGs. The majority of biological processes affected by thiram were metabolic. Kyoto Encyclopedia of Genes and Genomes pathway enrichment analysis yielded terms related to reproduction, such as steroid biosynthesis and steroid hormone biosynthesis. Quantitative real-time polymerase chain reaction validation results were in line with sequencing data for ten DEGs. The study results improve our current understanding of the effects of thiram exposure in ZF.

## 1. Introduction

Pesticide toxicity is a major threat to people and ecosystems worldwide. Pesticide usage has considerably increased in the previous decade in a bid to improve agricultural productivity and quality through the control of pests, fungi, and weeds [1,2]. In particular, fungicides are essential for crop protection, as they prevent fungal growth [3]. Their increasing use has led to increased environmental contamination and concerns regarding their effects on the environment as well as on human health [2,3,4]. Dithiocarbamate (DTC) fungicides were shown to be teratogenic, mutagenic, carcinogenic, and neurotoxic in animal and fish studies, possibly sharing a similar mechanism of toxicity [5,6,7,8]. Thiram is a DTC fungicide that is widely used to protect fruits, vegetables, ornamentals, and turf from pests [9]. It is also used as an accelerator and vulcanizing agent in the rubber industry [10]. Under field conditions, residual levels of thiram in the water of palm oil nurseries were found to range between 0.27 and 2.52 mg/L [11].

Thiram is listed as a category 1 potential endocrine disruptor by the European Union. It exerts reproductive and developmental damage in animal models [5,9]. The toxicological consequences of thiram in humans include thyroid problems and hepatotoxicity [12,13]. Among the adverse effects reported in thiram-exposed humans, asthenia, epistaxis, myocardiodystrophy, tachycardia, skin lesions, chest pain, eye irritation, and cough are also included. Thiram causes neurotoxic effects in animals, including lethargy and decreased motor activity [9]. Hamsters treated with thiram (125 mg/kg maternal dose) exhibited fused ribs, an underdeveloped spine and skull, as well as abnormal cardiac function [14]. Furthermore, the oral administration of thiram during pregnancy in mice resulted in increased resorption and fetal malformations, such as micrognathia, cleft palate, twisted ribs, and deformed bones [15], while thiram injection in pregnant rats slowed skull bone hardening in their offspring [9]. Syrian hamsters treated with thiram during pregnancy showed higher resorption rates, more terata, and smaller fetuses [5]. Thiram is known to prevent oviposition when added to the diets of quail, chickens, and partridges [16]. Acute toxicity studies of thiram in *Daphnia magna* revealed that it induces oxidative damage [17]. In *Cyprinus carpio*, it caused an increase in muscle and blood glucose levels in parallel to the depletion of liver and muscle glycogen [7]. According to the Pesticide Properties Database, thiram is toxic to *D. magna* (48 h EC_50_: 0.139 mg/L) and *Oncorhynchus mykiss* (96 h LC_50_: 0.171 mg/L) [18]. During embryonic development, thiram-exposed zebrafish (ZF) exhibited abnormal spinal curvature, thyroid dysfunction, and abnormal craniofacial development [6,19]. Other developmental thiram-associated defects in ZF embryos included growth retardation, heart malformations, hatch inhibition, and body curvature [20]. Despite multiple reports of thiram toxicity, the underlying molecular mechanisms are complex and remain elusive.

Next-generation sequencing has gained popularity in light of its capacity for the high-throughput analysis of complex biological phenomena [21]. Owing to their size, affordability, and ease in maintenance, ZF are considered excellent aquatic vertebrate model organisms, and various aspects of their development are studied in toxicity research [22]. The ease of genetic engineering in ZF further renders them excellent models for toxicity research, including sequencing and other omics analyses [23]. Transcriptome analysis has been successfully performed for the study of molecular processes underlying pesticide-induced toxicity and associated developmental deformities in ZF [23]. Importantly, toxicity results in ZF correlate well with those in mammals [24], highlighting the former as a valuable model.

In the present study, we aimed to explore the molecular responses to thiram exposure through a transcriptomics approach, including differential expression and pathway enrichment analyses. To this end, we performed high-throughput RNA-sequencing of thiram-treated ZF embryos. The current findings provide insights into signaling pathways concomitantly disrupted, potentially giving rise to developmental abnormalities in thiram-treated ZF.

## 2. Results

### 2.1. Developmental Toxicity Study

**Figure 1:**
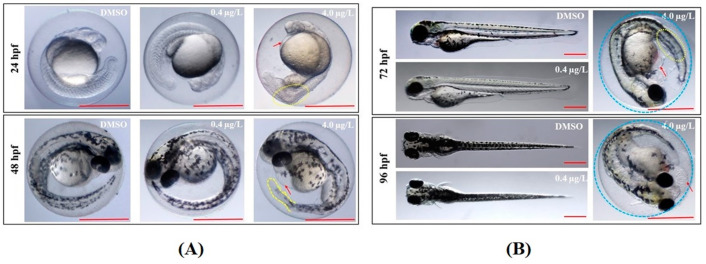
Representative zebrafish images showing malformations induced at specific thiram doses. (**A**) Upper panel (24 hpf) and lower panel (48 hpf); (**B**) upper panel (72 hpf) and lower panel (96 hpf). Yellow dotted ovals, abnormal somites; red arrows, pericardial edema; blue dotted circles, unhatched embryos; yellow dotted line, abnormal tail shape. Scale = 0.5 mm.

While phenotypes in the low-dose (0.4 µg/L) thiram-treated ZF (LTZF) group were similar to those of embryos and larvae of the dimethylsulfoxide (DMSO)-treated ZF (DTZF) group, several developmental abnormalities were noted in the high-dose (4.0 µg/L) thiram-treated ZF (HTZF) group (Table 1). These included somite deformities, delayed retinal pigmentation, body axis curvature, pericardial edema (PE), and yolk sac edema. Among the most noticeable abnormalities in the HTZF group, the inhibition of hatching, with no embryos hatching at 72 h post-fertilization (hpf) and less than 5% embryos hatching at 96 hpf (Table 1), was observed. The developmental anomalies observed at 24, 48, 72, and 96 hpf are indicated in Figure 1.

**Figure 2:**
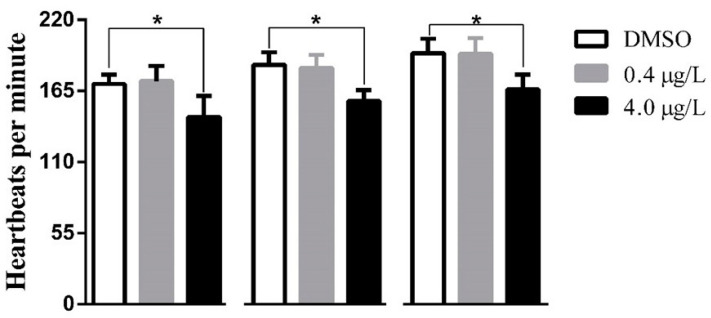
Bar graph showing mean heartbeats per minute observed at 48, 72, and 96 hpf. Results are presented as mean ± standard deviation (*n* = 3). Unpaired Student’s *t*-test was performed for statistical analysis. * statistically significant (*p* < 0.05).

ZF exhibit average heartbeats of 150–200 beats per min (bpm) between 30 and 60 hpf at 28 °C [25]. Thiram affected ZF cardiac function at a dose of 4.0 µg/L. DMZF had mean heart rates of 170.4 ± 7.1 (48 hpf), 185.2 ± 9.8 (72 hpf), and 194.2 ± 11.3 (96 hpf) bpm (Figure 2). LTZF exhibited similar mean heart rates of 172.6 ± 11.8 (48 hpf), 182.8 ± 10.2 (72 hpf), and 193.6 ± 12.4 (96 hpf) bpm. However, HTZF showed significantly lower heart rates than did DTZF (Video S1), at 145.0 ± 16.1 (48 hpf), 157.2 ± 8.4 (72 hpf), and 166.2 ± 11.5 (96 hpf) bpm. There was a high incidence of PE in the HTZF group (Table 1). At 24 hpf, 12.8% of ZF had edema symptoms, with PE increasing to 32.2% at 72 hpf. Hyperemia (Figure 1) and blood flow abnormalities (data not shown) were also evident in the HTZF group.

**Figure 3:**
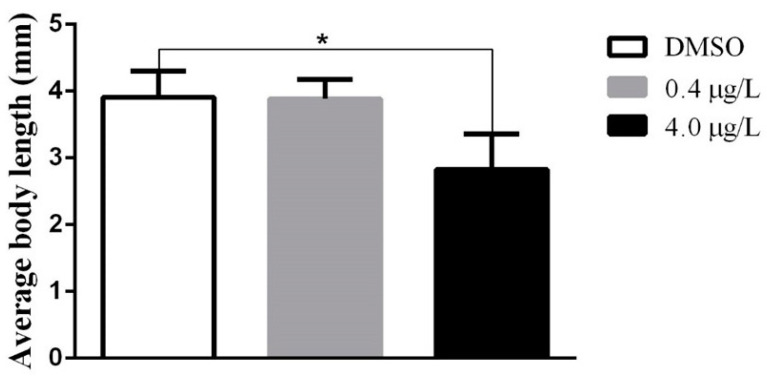
Bar graph showing the average body length at 144 hpf. Results are presented as mean ± standard deviation (*n* = 3). Unpaired Student’s *t*-tests were performed to assess significance. * statistically significant (*p* < 0.05).

**Figure 4:**
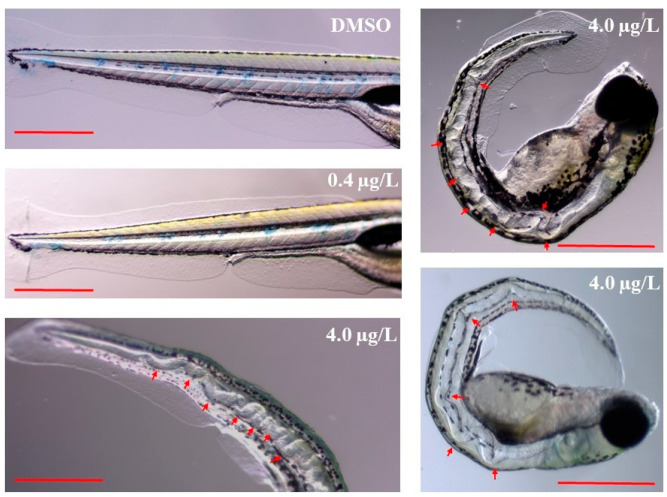
Representative images showing the notochord of the zebrafish at 144 hpf. Red arrows indicate the wavy distortions of notochords; Scale = 0.5 mm.

Thiram affected the overall growth of ZF (Figure 3). The mean growth of larvae in the LTZF group was similar to that of the DMZF group larvae, while HTZF had a shorter body length than the DMZF (27.74% shorter). Mean body lengths in the DMZF, LTZF, and HTZF groups at 144 hpf were 3.91 ± 0.39, 3.88 ± 0.29, and 2.82 ± 0.53 mm, respectively. Normal spines were noted in both the DMZF and LTZF groups, as opposed to spinal curvatures in the HTZF group (Table 1 and Figure S1), with wavy distortions of the notochords (Table 1 and Figure 4).

### 2.2. Whole-Transcriptome Analysis

**Figure 5:**
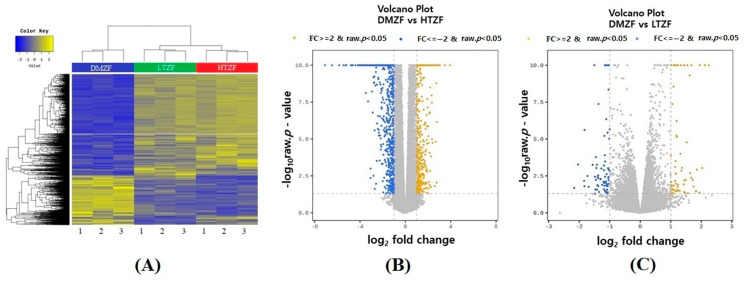
(**A**) Heat map of differential expression between DMZF, LTZF, and HTZF. Two-way hierarchical clustering heat map using the z-score for normalized values (log2-based). (**B**) Volcano plot of dimethylsulfoxide-treated zebrafish (DMZF) vs. 4.0 µg/L thiram-treated fish (HTZF). (**C**) Volcano plot of DMZF vs. 0.4 µg/L thiram-treated zebrafish (LTZF). Each blue colored dot indicates a downregulated gene (FDR < 0.05 and FC ≤ −2), and each yellow dot indicates an upregulated gene (FDR < 0.05 and FC ≥ 2).

Transcriptome analysis was performed (DMSO (*n* = 3), thiram 0.4 µg/L (*n* = 3), and thiram 4.0 µg/L (*n* = 3), 96 hpf) to comprehensively assess the molecular response to thiram exposure in ZF. Table 2 shows sequencing data from Illumina. Illumina-based sequencing data returned an average of 71,928,169 clean reads for DMSO-treated samples, 78,526,392 clean reads for 0.4 µg/L-treated samples, and 75,297,512 clean reads for 4.0 µg/L-treated samples. Over 98% of clean reads satisfied the Q20 threshold, and over 94% satisfied the Q30 threshold. An average of 86.95% clean reads were unequivocally mapped to the ZF reference genome (GRCz11). A heat map of two-way hierarchical clustering analysis with the z-score obtained from the normalized value (log2-based) is illustrated in Figure 5A. Using the *p*-value and fold change of each transcript, volcano graphs were constructed to show the overall distribution of transcripts as well as to determine differentially expressed genes (DEGs) under thiram treatment (Figure 5B,C). Cut-off values for DEG selection were a 2-fold change and a corrected false discovery rate (FDR) *p*-value of 0.05. A total of 1754 DEGs were obtained in the HTZF group, of which 70.80% (1242) were downregulated and 29.19% (512) upregulated (Figure S2). Only 138 DEGs were noted in the LTZF group (Figure S2). The top upregulated (FC > 5) genes in the HTZF group were those that encoded hemoglobin, alpha embryonic 5 (hbae5), D-amino-acid oxidase, tandem duplicate 1 (dao.1), suppressor of cytokine signaling 3a (socs3a), titin-cap (telethonin) (tcap), si:dkey-21p1.3, transcript variant X2 (si:dkey-21p1.3), si:ch73-265d7.2 (si:ch73-265d7.2), im:7152855, transcript variant X1 (im:7152855), complement component C6-like, transcript variant X1 (LOC798694), egl-9 family hypoxia-inducible factor 3 (egln3), transglutaminase 2, like, transcript variant X1 (tgm2l), Jun dimerization protein 2b (jdp2b), podocan-like protein 1 (LOC101882092), heat shock cognate 70-kd protein-like (hsp70l), cocaine- and amphetamine-regulated transcript protein-like (LOC557301), v-fos FBJ murine osteosarcoma viral oncogene homolog Ab (fosab), ankyrin repeat domain 37 (ankrd37), short chain dehydrogenase/reductase family 42E, member 2, transcript variant X2 (sdr42e2), and somatolactin beta (smtlb) (Table S1). The top downregulated (FC < −15) genes in the HTZF group encoded elastase 3 like (ela3l), elastase 2 (ela2), syncollin, tandem duplicate 2 (sycn.2), chitinase, acidic.2 (chia.2), elastase 2 like (ela2l), lactase-phlorizin hydrolase (LOC559107), carboxypeptidase A1 (pancreatic) (cpa1), amine oxidase, copper containing 1 (aoc1), UDP glucuronosyltransferase 5 family, polypeptide A2, (ugt5a2), UDP glucuronosyltransferase 5 family, polypeptide A4 (ugt5a4), solute carrier family 26, member 3, tandem duplicate 2 (slc26a3.2), zonadhesin, transcript variant X1 (LOC100147904), cytochrome P450, family 2, subfamily N, polypeptide 13 (cyp2n13), meprin A, alpha (PABA peptide hydrolase), tandem duplicate 1 (mep1a.1), mucin 2.1 (muc2.1), purine nucleoside phosphorylase 4b (pnp4b), glucokinase (hexokinase 4) (gck), alanyl (membrane) aminopeptidase b (anpepb), and 2-epi-5-epi-valiolone synthase (eevs) (Table S1).

### 2.3. Gene Ontology (GO) Enrichment Analysis

**Figure 6:**
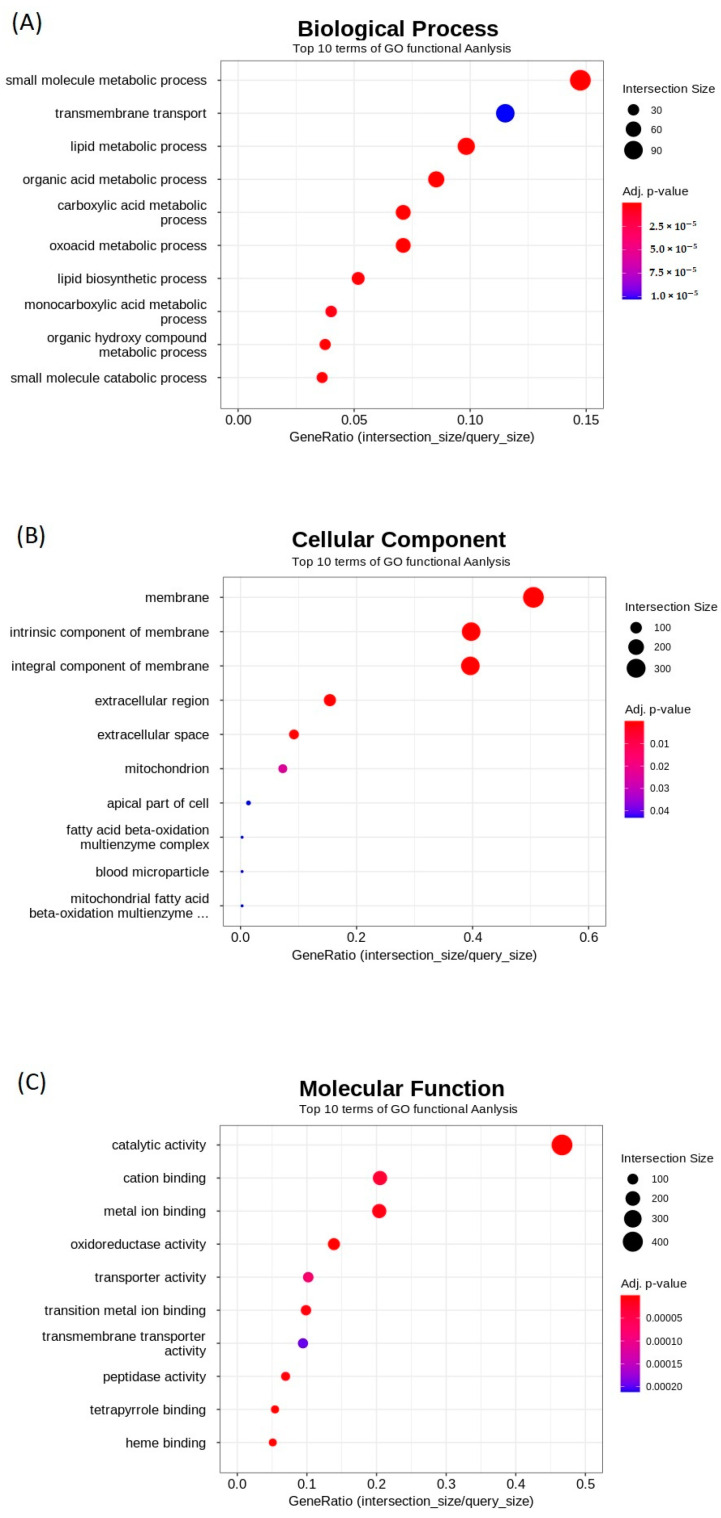
Top 10 terms of GO functional enrichment analysis. Biological process (BP) (**A**), cellular component (CC) (**B**), and molecular function (MF) (**C**) terms associated with differentially expressed genes.

Significant DEGs were classified into three functional categories as per GO enrichment analysis, including biological process (BP), cellular component (CC), and molecular function (MF). Figure 6 depicts the top ten enriched GO terms in each category following high-dose thiram therapy (HTZF group). Metabolic processes, biosynthetic processes, and catabolic processes were among the 10 most enriched terms for “BP” (Figure 6A). With regard to the top 10 enriched terms for “CC”, most were related to the membrane and extracellular region (Figure 6B). Most MF terms were associated with oxidoreductase, ion binding, and catalytic activity (Figure 6C).

### 2.4. Kyoto Encyclopedia of Genes and Genomes (KEGG) Pathway Enrichment Analysis

DEGs were subjected to KEGG pathway enrichment analysis to determine signaling pathways regulated in response to thiram treatment. A total of 77 signaling pathways were significantly enriched in the HTZF group (*p* < 0.05). These were categorized into six subgroups. The metabolism subcategory was at the top, with 52 signaling pathways, followed by cellular processes, with 11 signaling pathways. Organismal systems and environmental processing included six pathways each, while human diseases and genetic processing consisted of one pathway each (Figure S3). A total of 70 signaling pathways were enriched in the LTZF group (*p* < 0.05), grouped into six categories. The metabolism subcategory was once again the largest, with 44 signaling pathways, followed by cellular processes with eight signaling pathways. The organismal systems, environmental processing, genetic information processing, and human diseases subcategories included 6, 5, 1, and 1 signaling pathways, respectively (Figure S3).

**Figure 7:**
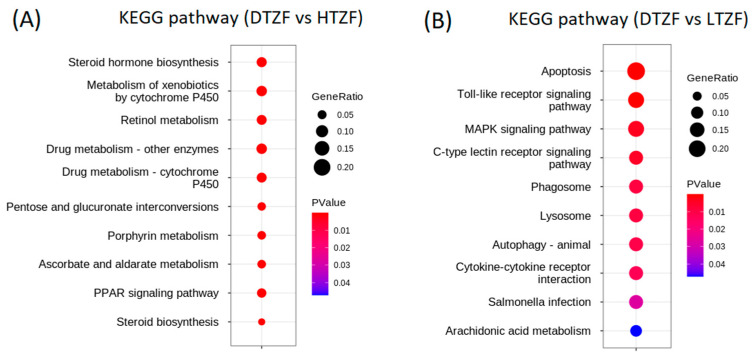
Top 10 KEGG pathways in the (**A**) HTZF (4.0 µg/L thiram-treated ZF) and (**B**) LTZF (0.4 μg/L thiram-treated ZF) compared with the DMZF group (dimethylsulfoxide-treated ZF).

In the HTZF group, the top 10 enriched KEGG pathways (*p* ≤ 0.01) included steroid hormone biosynthesis (dre00140), metabolism of xenobiotics by cytochrome P450 (dre00980), retinol metabolism (dre00830), drug metabolism—other enzymes (dre00983), drug metabolism—cytochrome P450 (dre00982), pentose and glucuronate interconversions (dre00040), prophyrin metabolism (dre00860), ascorbate and aldarate metabolism (dre00053), PPAR signaling pathway (dre003320), and steroid biosynthesis (dre00100) (Figure 7A). Table 3 lists up- and downregulated DEGs in the 10 major KEGG pathways. The top 10 enriched KEGG pathways (*p* ≤ 0.05) of the LTZF group included apoptosis (dre04210), toll-like receptor signaling pathway (dre04260), MAPK signaling pathway (dre04010), C-type lectin receptor signaling pathway (dre04265), phagosome (dre04145), lysosome (dre04142), autophagy—animal (dre04140), cytokine–cytokine receptor interaction (dre04060), Salmonella infection (dre05132), and arachidonic acid metabolism (dre00590) (Figure 7B).

### 2.5. Quantitative Real-Time Polymerase Chain Reaction (qPCR) Validation of Transcriptome Data

The transcript levels of ten randomly selected DEGs with up- or downregulation tendencies were examined using qPCR. All genes presented the same expression patterns in agreement with RNA-seq data (Table 4).

## 3. Discussion

Given the extreme toxicity of pesticides, it is crucial to obtain a comprehensive understanding of the molecular mechanisms activated in response to pesticide exposure in off-target organisms. Pesticides exert considerable toxicity, capable of disrupting cellular homeostasis [26]. Herein, we employed next-generation RNA-sequencing technology to explore the response to thiram exposure in ZF at the molecular level. To this end, developmental deformities were scored, and ZF samples were subjected to whole-transcriptome analysis. A wide spectrum of developmental deformities were noted in the HTZF group (Figure 1), with 1754 DEGs, including 512 upregulated and 1242 downregulated DEGs (Figure S1). Thus, 4.0 μg/L of thiram considerably disrupted cellular homeostasis, impacting the normal growth and development of ZF.

Thiram has been included in the European Union’s Category 1 list of endocrine disruptors based on extensive scientific evidence. Female rats treated with thiram exhibited a disrupted hormonal control of ovulation [27]. Thiram inhibited the rise in luteinizing hormone (LH) at a dose of 50 mg/kg in all rats tested [27]. Chen et al. recently reported that early thiram exposure disrupts endocrine function in ZF [19]. Furthermore, results from our laboratory showed that thiram affected the fecundity of ZF (data not shown). Interestingly, KEGG pathway enrichment analysis of DEGs obtained from the HTZF group revealed a significant enrichment of hormone-related signaling pathways, including steroid biosynthesis, steroid hormone biosynthesis, and retinol metabolism (Figure 7).

“Steroid hormone biosynthesis” was the top-ranked enriched pathway as per KEGG results for the HTZF group (Figure 7), indicating that the disruption of steroid hormone homeostasis is the major underlying mechanism of thiram toxicity in ZF. Steroid hormones are required for the initiation and maintenance of secondary sex characteristics, reproduction, and other bodily functions [28]. In the steroid hormone biosynthesis pathway, several UDP-glucuronosyltransferase (UGT) (21 genes) and cytochrome P450 (CYP) (five genes) genes were downregulated in response to thiram. UGT and CYP enzymes both have detoxification activity [29]. More specifically, UGT glucuronidates a variety of endogenous and exogenous substrates, while CYPs oxidize. In addition, UGTs are primarily involved in androgen signaling [30] and CYPs in estrogen signaling [31]. Several important genes, such as *srd5a2a* (steroid 5-alpha-reductase, alpha-polypeptide 2a), *had11b1la* (hydroxysteroid (11-beta) dehydrogenase 1-like a), and *comta* (catechol O methyltransferase a) were also downregulated in the HTZF group. SRD5A2 converts testosterone into the more potent dihydrotestosterone (DHT), an essential hormone in testicular development and spermatogenesis [32]. 11β-hydroxysteroid dehydrogenase is essential in fish spermatogenesis, as it is required for the conversion of 11β-hydroxytestosterone to 11-ketotestosterone (11-KT) and the conversion of cortisol to cortisone [33]. Catechol O methyl transferase is essential for reproduction and metabolizing estrogen as well as epinephrine, catecholamines, and norepinephrine [34]. Sex steroids permanently constitute the brain circuits responsible for reproductive behavior during fetal development, stimulating these specialized pathways when released from the gonads during puberty [35]. For example, the ZF male holding water contains 17 beta-diol, 5 alpha-androstane-3 alpha, and cholesterol, which act as ovulation inducers [36].

Another KEGG pathway enriched for the HTZF group was “steroid biosynthesis” (Figure 7). Human fertility is negatively impacted by genetic abnormalities that manifest as functional changes in proteins of the steroidogenic pathway [37]. Four genes known to play an essential role in cholesterol biosynthesis, namely, methyl sterol monooxygenase 1 (*msmo1*), lanosterol synthase (*lss*), emopamil-binding protein (*ebp*), and 7-dehydrocholesterol-Δ7 reductase (*DHCR7*), were downregulated in the HTZF group (Table 3). Msmo1 is the key enzyme in the post-squalene cholesterol biosynthetic pathway and removes a methyl group from C4-methyl sterols [38]. Lanosterol synthase is required for the cyclization of the first sterol, an essential step in cholesterol biosynthesis [39]. Emopamil-binding protein acts as a sterol isomerase in the pathway [40]. DHCR7 is required to complete the conversion of 7-dehydrocholesterol to cholesterol, as the final step [41]. Furthermore, cholesterol serves as a critical precursor for the biosynthesis of steroid hormones, which in turn regulate reproductive pathways and the development of secondary sexual characteristics, and de novo defects in cholesterol synthesis impact fertility and embryo viability [37].

“Retinol metabolism” was also enriched in the KEGG pathway analysis of HTZF (Figure 7). Retinoids play an essential role in various physiological processes, including reproduction, growth and development, vision, and the immune system [42,43]. Exposure to diethylaminobenzaldehyde (DEAB), an inhibitor of retinoid synthesis, or intake of a low-retinoid diet resulted in ZF producing 70 to 90% fewer eggs [42]. Retinoic acid deprivation at the embryonic stages of ZF development resulted in microphthalmia before birth [42]. Embryos deprived of retinoic acid also developed severe PE, smaller eyes, yolk sac edema, and visual disturbances [42]. As the HTZF group exhibited similar developmental anomalies (Figure 1), it is conceivable that some of these were due to defects in retinol metabolism.

The next protein downregulated in response to thiram was Zondhesin (ZAN). ZAN is a multi-domain sperm protein essential in the binding interaction of sperm and eggs during reproduction [44]. Angiotensin I-converting enzyme 2 (ACE2) was downregulated about 16-fold in the HTZF group. According to Keskus et al., ace2 expression is crucial for ZF embryonic development, with in silico analysis of transcriptome datasets revealing that ace2 expression predominantly increases from 3 days post fertilization (dpf) to 4 dpf [45]; this result provides support for its role in ZF embryonic development.

All fish in the HTZF group displayed wavy notochord distortions and skeletal deformities (Figure 4). The results are in line with those of Chen et al., who found that ZF treated with thiram exhibited wavy distortions of the notochords [19]. The findings are consistent with those in previous reports of body axis curvatures and notochord distortions in thiram-treated ZF [20]. Inhibition of copper-dependent lysyl oxidases was previously shown to lead to abnormal notochords in ZF embryos [46], while the addition of copper restored the wavy notochord phenotypes in ZF [47]. It is therefore possible that the wavy notochord phenotype seen after thiram treatment (Figure 5) is due to the synergistic effects of thiram-mediated lysyl oxidase suppression (Table 3) and copper ion chelation. Downregulated mucins (mucin 2.1, 16.76-fold, and mucin 13b, 5.66-fold) could equally explain the skeletal deformities observed in HTZF, since mucin knockout mice have a decreased bone mass and increased osteoblast mineralization activity [48]. The expression of three genes involved in cholesterol biosynthesis (*msmo1, lss*, and *ebp*) was downregulated in the HTZF group (Table 3), with deficiencies in genes required for cholesterol biosynthesis inducing bone defects. Moreover, *msmo1* and *lss* are essential in bone formation, and mutations of these genes in prehypertrophic chondrocytes cause severe abnormalities in bone formation due to cholesterol deficiency and intermediate sterol accumulation [49]. Mutation of the *ebp* gene in humans is associated with craniofacial and skeletal abnormalities, as well as ocular or visual defects [50]. Undoubtedly, further research is needed to better understand the causes of these abnormalities in relation to thiram.

Another group of enzymes that were significantly downregulated in the HTZF group includes chitinase, acidic.1 (chia.1), chitinase, acidic.2 (chia.2), chitinase, acidic.5 (chia.5), and chitin synthase 1 (chs1). Chitin is a structural polysaccharide that is endogenously synthesized in fish, particularly during the early phases of development. Knockdown of chs1 in developing ZF embryos resulted in a significant reduction in chitin signals within the gut [51]. Mammalian chitinases/chitinase-like proteins have been linked to various innate and acquired immunological responses, as well as tissue remodeling and fibrosis induced by both chitinous and non-chitinous stimuli. Chitinase(s) are also known to be involved in normal development of the trunk and tail in ZF [52].

There was a 17-fold downregulation in the expression of meprin A, alpha, tandem duplicate 1 (mep1a.1) in response to thiram. Merpins are expressed in a variety of tissues in ZF, including the gills, gut, liver, skin, kidneys, brain, and heart [53]. According to Schütte et al., meprins plays essential roles in embryonic cell differentiation and proliferation as well as angiogenesis [54]. Meprin 1 knockdown ZF larvae displayed a dilated pericardium or deformed tissues in the trunk and tail due to issues with cell differentiation. Therefore, cardiac abnormalities observed with thiram-treated ZF are possibly due to the reduction of meprin levels.

There was a more than 5-fold downregulation in the expression of nine tRNAs (tRNA-Asp, tRNA-Glu, tRNA-Pro, tRNA-Ala, tRNA-Leu, tRNA-Trp, tRNA-Val, tRNA-Thr, and tRNA-Cys) in the HTZF group compared with that in the DMZF group. tRNAs are assumed to be exclusively involved in translation, as they are originally known for carrying amino acids during protein synthesis. However, recent studies have demonstrated that tRNAs are essential in numerous other processes, such as the control of transcription and translation, post-translational modifications, stress reactions, and diseases [55]. For example, Revendro et al. reported that tRNA dysregulation in ZF causes reduced embryo viability and increased malformations, and postulated that tRNA dysregulation promotes protein aggregation and genomic instability [56]. Reactive oxygen species accumulation, nuclear and mitochondrial DNA damage, and mitochondrial network alterations have also been observed in ZF embryos with tRNA mutations resulting in the incorrect incorporation of serine (Ser) [56]. The expression of several factors known to mediate global protein biosynthesis [57,58], such as translation initiation factors (eIF4A1a and IF21) and termination factors (eTF1a), was also downregulated in the HTZF group (File S1). It is possible that disruptions in protein synthesis were responsible for the growth retardation and other abnormalities seen in the HTZF group (Figure 3). Interestingly, transcriptome results from the present study (thiram treatment) and our earlier study (phosmet treatment) suggested the disruption in protein synthesis as one of the main mechanisms of toxicity [23]. However, while phosmet decreased the expression of several aminoacyl synthases that ligate tRNAs to their corresponding amino acids, thiram downregulated the expression of several tRNAs, suggesting that pesticides dysregulate protein synthesis through different mechanisms.

The expression of some miRNAs, such as miR-126a (8.4 fold), miR-181a-1, (8.37 fold), and miR-133b (5.03 fold) was downregulated in the HTZF group. MiR-126 is highly enriched in vascular endothelium and is crucial for vasculogenesis, angiogenesis, and endothelial inflammation [59]. MiR-126a silencing impaired the formation of parachordal lymphangiogenic budding from the posterior cardinal vein and thoracic duct of ZF embryos [60]. There is evidence that miR-181 is essential in embryonic development, cell proliferation, autophagy, apoptosis, immune response, and mitochondrial function [61]. The expression of miR-133, crucial in cardiac diseases and development, was decreased during cardiac regeneration, enhancing cardiomyocyte regeneration [62]. In ZF, downregulation of miR-133 expression alters the muscle gene expression pattern and leads to actin disorganization during sarcomere assembly [63].

Early ZF embryonic development is exclusively dependent on the egg yolk for nutrition [64]. Thus, hatching delay or inhibition is deleterious [65]. ZF embryos start hatching at 35 hpf, and almost all hatch at 96 hpf under ideal conditions [66]. Hatch failure was the most significant deformity observed in ZF after treatment with thiram (Table 1). In the HTZF group, almost all embryos remained in their chirons. These results are consistent with findings from previous studies [19,20]. As ZF embryos did not hatch in the presence of other DTCs, such as maneb [67] and ziram [68], it is conceivable that the inhibition of hatching is among the most important toxic effects of DTCs on ZF. The potential to prevent hatching could be explained by either a decrease in the larva’s ability to destroy the chorion or by the suppression of proteins required for chorion digestion. The downregulation of expression of tetraspanins (tspan 34 and tspan 13a) (Table 3), required for chorionic digestion in ZF [69], and/or morphological defects, such as tail deformities and spinal curvature, impair the embryo’s capacity to rupture the chorion [70,71], possibly causing the hatching failure observed in the HTZF. Because hatching is a critical step of reproduction, hatching delays have a considerable negative impact on populations and ecology [72].

Transcriptome analysis of the LTZF group revealed 138 DEGs compared to the DTZF group (Figure S2), suggesting that thiram elicits molecular responses even at low concentrations (0.4 µg/L). Despite the lack of an apparent phenotype in the LTZF group (Table 1; Figure 1, Figure 2, Figure 3, and Figure 4), we cannot completely rule out the possibility that lower-dose exposure may have long-term consequences on ZF development.

Understanding the mechanisms activated during pesticide poisoning in ZF is expected to provide clues for the analogous responses in humans owing to the high degree of genetic homology [73]. The ZF model allows for faster in vivo toxicity evaluation than mammalian tests [74]. Animal toxicological studies often reveal consequences that require additional, costly, and time-consuming research into the exact cause. Combined with transcriptome analysis, toxicity screening approaches employed in ZF allow for the identification of off-target effects as well as for the study of putative underlying mechanisms. ZF models are essential for the identification of the safest candidates for pesticide production and for determining optimal approaches to treat pesticide poisoning. The current work elucidated the molecular response to pesticide toxicity, demonstrating the feasibility of state-of-the-art methods for this purpose.

## 4. Materials and Methods

### 4.1. Ethics Statement

All animal tests were performed in compliance with the standards of the care and use of laboratory animals and were approved by the Animal Ethics Committee of the National Institute of Agricultural Sciences, Rural Development Administration, Republic of Korea (NAS-202102).

### 4.2. ZF Husbandry and Embryo Selection

ZF (*Danio rerio*, AB strain) were cultured at the fish breeding facility of the National Institute of Agricultural Sciences in South Korea in order to obtain embryos. Fish cultures were maintained as previously described [70,71]. They were kept in a glass tank (50 L) with dechlorinated water and a continuously circulating filter method. The breeding facility was kept at a constant temperature of 25 ± 1 °C and a photoperiod of 14 h light/10 h dark. The fish received one of three diets, including bloodworm (Hikari Bio-pure, Himeji, Japan), live Artemia (INVE Aquakultur, Dendermonde, Belgium), and dry flake diet (Top Meal, Tabia, Korea), at least twice a day. Collection and washing techniques for ZF embryos were previously described [20,23]. Males and females in a 1:1 ratio were mated in the dark for 10 h. Once the light was turned on for 30 min, the embryos were harvested and washed repeatedly with E3 medium to completely remove debris from their surface. Fertilized embryos were selected at 2 hpf and used for the pesticide treatment.

### 4.3. Thiram Treatment and Scoring of Developmental Deformities

Thiram (98.4%, Sigma-Aldrich, St. Louis, MO, USA) was dissolved in DMSO (Sigma-Aldrich). Thiram stocks were aliquoted and stored at –20 °C until use. Test doses were determined according to a previous study [20]. Two test doses were chosen, with the higher thiram dose of 4.0 µg/L being the average EC_50_ value for ZF, which would ensure that the most deformities were induced in the collected embryos. The other test dose, 0.4 µg/L, was a 10-fold dilution of the higher test dose. The DMSO concentration in all treatment groups was 0.01%. For the developmental toxicity assay, two 24-well plates, each containing 20 embryos, were used for each test condition. The experiments were repeated three times. Chemical exposure was started after 2.0 hpf, and the experimental conditions for chemical treatment, deformity assessment, and the calculation of percentage deformed embryos were similar to those previously described [20].

### 4.4. Heartbeat Survey

Heartbeat studies were performed under a stereomicroscope (Stemi 508, Zeiss, Germany) at three time intervals (48, 72, and 96 hpf) at 26 ± 1 °C. Heartbeats were counted for 20 s, converted to beats per min, and reported. The experiment was performed three times, and each time the heartbeats of 10 embryos were evaluated (*n* = 30 embryos).

### 4.5. Touch-Evoked Escape Response (TEER)

As previously reported [23], TEER was performed at 144 hpf. The TEER was measured by carefully touching the ZF’s head and tail with a thin (1 mm) flexible wire. The experiment was performed three times, and each time, 10 embryos were evaluated (*n* = 30 embryos).

### 4.6. Body Length Survey

Body length studies were performed at 144 hpf using a stereomicroscope to take pictures. OptiView 3.7 software (Korealabtech, Seongnam, Republic of Korea) was used to calculate body length (from the tip of the mouth to the end of the caudal fin). The experiment was performed three times, and each time, the body length of 10 embryos was evaluated (*n* = 30 embryos).

### 4.7. Statistical Analysis

An unpaired *t*-test (Prism 5.0, GraphPad, San Diego, CA, USA) was used to determine significant differences between samples in the analyses of developmental deformities, heart rate, and body length. The *p* values ≤ 0.05 were considered as statistically significant.

### 4.8. RNA-Sequencing

Samples were collected in triplicate for transcriptome analysis and qPCR validation. Samples containing at least 30 embryos of the 4.0 µg/L thiram-treated, 0.4 µg/L thiram-treated, and DMSO-treated groups were snap-frozen in liquid nitrogen and stored at −80 °C until use. Total RNA extraction, RNA quantification, RNA integrity assessment, RNA library preparation, and cDNA library preparation were performed according to previously described protocols [23]. Paired-end (2 × 100 bp) sequencing was performed on the NovaSeq platform (Illumina, Inc., San Diego, CA, USA).

### 4.9. DEG Selection

By comparing DMSO- and thiram-treated groups, DEGs were selected using nbinomWaldTest and DESeq2. The DEG selection criteria were a *p*-value of 0.05 and a cut-off of the absolute log2-fold change of 2. The acquired DEGs were subjected to further analyses.

### 4.10. DEG Analysis

Pathway enrichment analysis of DEGs was performed using KEGG Pathways Enrichment Analysis (http://www.kegg.jp/kegg/pathway.html, accessed on 10 October 2022). The GO enrichment analysis of DEGs was conducted using the g:Profiler tool (https://biit.cs.ut.ee/gprofiler/, accessed on 14 October 2022).

### 4.11. Quantitative Reverse Transcription-Polymerase Chain Reaction Validation

The total RNA content was extracted from three samples per test condition using the TRIzol reagent (Bioneer, Daejon, South Korea). The Nanodrop 2000 was used to examine the RNA (Thermo Fisher Scientific, MA, USA). To reverse-transcribe RNA into cDNA, the ReverTra Ace™ qPCR RT master mix (Toyobo, Osaka, Japan) was used. TOPreal^TM^ qPCR 2X premix (Enzynomics, Daejon, Republic of Korea) and CFX96 Dx real-time PCR detection equipment (Biorad, CA, USA) were utilized to perform qPCR. The Ct values of all samples were obtained in triplicates. Average Ct values were used to calculate the gene expression fold change. As a housekeeping gene, β-actin was employed. For the expression analysis, the 2^−ΔΔCT^ approach was used. The oligoprimers utilized for qPCR are listed in Table S2.

## 5. Conclusions

As thiram is widely used in agriculture, it is important to understand its toxicity to aquatic life. The current results show that thiram extensively influences gene expression, affecting the growth, reproduction, and behavior of the fish. More specifically, thiram disrupts ZF reproduction by interfering with pathways involved in steroid hormone biosynthesis. These findings also provide substantive insights into the molecular pathways altered in response to thiram and thus causing developmental toxicity in ZF. Further research into thiram disposal in aquatic environments is required.

## Figures and Tables

**Table 1:** Proportions of developmental deformities scored after 24, 48, 72, 96, and 144 h of thiram exposure.

Time	Deformity (%)	DMZF (DMSO)	LTZF (0.4 µg/L Thiram)	HTZF (4.0 µg/L Thiram)
24 hpf	Edema symptoms	1.71 ± 1.48	1.71 ± 2.96	12.80 ± 5.47
	Abnormal somites	1.66 ± 1.44	3.40 ± 1.50	50.83 ± 6.29 *
48 hpf	Low retina pigment	3.42 ± 1.59	2.59 ± 2.56	31.84 ± 8.07 *
	Abnormal tail blood flow	3.42 ± 1.59	1.71 ± 2.96	22.16 ± 4.45 *
72 hpf	Unhatched embryos	0.88 ± 1.52	3.47 ± 1.44	99.05 ± 1.65 *
	Pericardial edema	1.69 ± 1.46	6.08 ± 1.41	32.20 ± 6.38 *
	Yolk sac edema	0	2.56 ± 2.56	37.10 ± 5.49 *
96 hpf	Unhatched embryos	0.88 ± 1.52	1.78 ± 1.54	99.02 ± 1.70 *
144 hpf	Body curvature	0	0	100.00 *
	Wavy distortions of notochord	0	0	99.02 ± 1.70 *
	Abnormal touch response	0	0	46.10 ± 6.95 *

Data are shown as the mean ± standard deviation of three independent experiments (40 embryos in each experiment (body curvature, wavy distortions of notochord, and abnormal touch response 10 embryos in each experiment)). Unpaired Student’s *t*-test was performed for assessing statistical significance. * Statistically significant (*p* < 0.05).

**Table 2:** Sequencing data from transcriptome analysis and quality filtering.

Samples	Total Reads	Clean Reads	Mapped Reads	Mapped Rate (%)	GC (%)	Q20 (%)	Q30 (%)
DMZF-1	63,609,300	62,755,930	55,712,761	88.78	43.38	98.16	94.43
DMZF-2	74,512,768	73,530,624	65,089,037	88.52	43.07	98.22	94.65
DMZF-3	80,690,306	79,497,952	70,496,653	88.68	43.37	98.02	94.11
LTZF-1	80,299,462	79,177,492	70,038,625	88.46	43.65	98.18	94.57
LTZF-2	75,717,900	74,610,672	66,263,993	88.81	43.82	98.04	94.17
LTZF-3	78,087,892	76,903,732	67,627,127	87.94	43.11	97.96	93.97
HTZF-1	77,703,262	76,496,736	64,893,724	84.83	43.35	98.05	94.27
HTZF-2	76,694,784	75,460,228	63,576,050	84.25	43.23	97.99	94.13
HTZF-3	74,987,914	73,935,572	61,222,810	82.81	43.68	98.18	94.55

DMZF, dimethylsulfoxide-treated zebrafish; LTZF, Low-dose (0.4 μg/L) thiram-treated zebrafish; HTZF, High-dose (4.0 μg/L) thiram-treated zebrafish; GC, guanine–cytosine; Q30, Phred quality score 30; Q20, Phred quality score 20.

**Table 3:** List of significant DEGs for each enriched KEGG pathway in the HTZF group.

Term	Genes	*p*-Values	FDR
Metabolism of xenobiotics by cytochrome P450	*ugt5a2, ugt5a4, ugt5a1, gsto1, ugt1a1, ugt1a7, ugt5b3, ugt1ab, zgc:77938, gstp2, ugt5d1, ugt5b4, ugt2a1, ugt2a4, ugt1a4, ugt1a5, ugt1a2, ugt1a6, gsta.1, ugt5b1, ugt1b2, ugt2b1, mgst1.2, cbr1, gstt1a, cbr1l, akr7a3, ugt2b3, ugt5g1, hsd11b1la, dhdhl, gsto2, ephx1, gstt2, gstt1b, adh8b*	3.0421 × 10^−40^	4.2893 × 10^−38^
Drug metabolism—cytochrome P450	*gstt18, gstt33, gstt16, gstt9, gstt17, gstt23, gstt31, gstt7, gstt5, gstt11, gstt32, gstt19, gstt21, gstt20, gstt29, gstt27, gstt22, gstt26, gstt6, gstt30, gstt24, gstt25, gstt8, gstt13, gstt14, gstt28, gstt15, gstt12, gstt10, gstt2, gstt3, gstt4*	1.2906 × 10^−34^	9.1369 × 10^−33^
Drug metabolism—other enzymes	*gstt52, gstt68, gstt50, gstt41, gstt51, gstt58, gstt66, gstt39, gstt45, gstt67, gstt53, gstt55, gstt54, gstt64, gstt47, gstt62, gstt69, gstt57, gstt61, gstt38, gstt65, gstt37, gstt59, gstt60, gstt40, gstt48, gstt56, gstt46, gstt63, gstt49, gstt42, gstt44, gstt43, gstt35, gstt36, gstt34*	2.9616 × 10^−34^	1.3919 × 10^−32^
Steroid hormone biosynthesis	*ugt5a2, ugt5a4, cyp3a65, ugt5a1, cyp3c3, ugt1a1, ugt1a7, cyp3c4, ugt5b3, cyp7a1, srd5a2a, ugt1ab, ugt5d1, ugt5b4, zgc:92630, ugt2a1, ugt2a4, ugt1a4, ugt1a5, ugt1a2, sult2st2, ugt1a6, dhrs11b, ugt5b1, ugt1b2, ugt2b1, hsd3b1, ugt2b3, ugt5g1, hsd11b1la, cyp7a1b, comta, hsd17b7*	5.2260 × 10^−34^	1.8421 × 10^−32^
Retinol metabolism	*ugt5a2, ugt5a4, si:ch1073-13h15.3, cyp3a65, ugt5a1, cyp3c3, bco1l, ugt1a1, ugt1a7, cyp3c4, ugt5b3, ugt1ab, zgc:77938, ugt5d1, ugt5b4, si:ch211-107o10.3, ugt2a1, ugt2a4, ugt1a4, ugt1a5, ugt1a2, ugt1a6, dgat1a, ugt5b1, ugt1b2, ugt2b1, retsat, ugt2b3, ugt5g1 adh8b, **lrata***	1.8112 × 10^−30^	5.1092 × 10^−29^
PPAR signaling pathway	*fabp6, cd36, fabp2, cyp7a1, fads2, cyp8b1, fabp1b.1, acsl5, cyp8b3, cyp8b2, si:ch211-113j14.1, acsl1b, acadm, acox1, slc27a4, cyp7a1b, zgc:101540, cpt2, slc27a2b, scp2a, si:dkey-91i10.3, acsbg1, hmgcs1, acadl, **aqp7**, **slc27a2a**, **LOC568656**, **plin1***	1.0274 × 10^−24^	2.4144 × 10^−23^
Ascorbate and aldarate metabolism	*ugt5a2, ugt5a4, ugt5a1, ugt1a1, ugt1a7, ugt5b3, ugt1ab, ugt5d1, ugt5b4, ugt2a1, ugt2a4, ugt1a4, ugt1a5, ugt1a2, ugt1a6, ugt5b1, ugt1b2, ugt2b1, ugdh, ugt2b3, ugt5g1, aldh7a1, aldh3a2b*	1.2422 × 10^−23^	2.5021 × 10^−22^
Pentose and glucuronate interconversions	*ugt5a2, ugt5a4, ugt5a1, ugt1a1, ugt1a7, ugt5b3, ugt1ab, ugt5d1, ugt5b4, ugt2a1, ugt2a4, ugt1a4, ugt1a5, ugt1a2, ugt1a6, ugt5b1, ugt1b2, ugt2b1, ugdh, ugt2b3, ugt5g1, dhdhl*	2.6641 × 10^−22^	4.6954 × 10^−21^
Porphyrin metabolism	*ugt5a2, ugt5a4, ugt5a1, ugt1a1, ugt1a7, ugt5b3, ugt1ab, ugt5d1, ugt5b4, ugt2a1, ugt2a4, ugt1a4, ugt1a5, ugt1a2, ugt1a6, ugt5b1, ugt1b2, ugt2b1, fech, blvra, ugt2b3, ugt5g1, **alas2***	8.1199 × 10^−22^	1.2721 × 10^−20^
Steroid biosynthesis	*cel.2, cel.1, msmo1, ebp, sc5d, nsdhl, cyp2r1, lss, cyp51, sqlea, hsd17b7, dhcr7, **soat2**, **cyp24a1***	1.3069 × 10^−17^	1.8427 × 10^−17^

FDR: false discovery rate, downregulated (normal text), upregulated (bold text).

**Table 4:** qPCR-based validation of ten randomly selected DEGs from transcriptome data.

Gene Symbol	Fold Change (RNA-seq)	Fold Change (qPCR) (Mean ± SD)
*hbae5*	15.26	31.01 ± 9.62
*socs3a*	8.22	7.99 ± 2.33
*Tcap*	7.69	6.35 ± 2.98
*egln3*	6.14	3.57 ± 3.24
*jdp2b*	5.73	4.67 ± 1.15
*Smtlb*	5.10	4.67 ± 2.07
*ela2*	−57.17	−47.72 ± 15.61
*chia.2*	−38.59	−25.56 ± 11.17
*aoc1*	−27.60	−3.21 ± 1.13
*ugt5a4*	−24.68	-22.54 ± 3.75

DEGs: differentially expressed genes, qPCR: quantitative real-time polymerase chain reaction, SD: standard deviation.

## Data Availability

Data are contained within the article or Supplementary Materials.

## Supplementary Materials

The following supporting information can be downloaded at: https://www.mdpi.com/article/10.3390/biology12020156/s1, Figure S1: Representative images showing spinal curves observed in high-dose (4.0 μg/L) thiram-treated zebrafish (HTZF); Scale = 0.5 mm. Figure S2: Numbers of up- and downregulated differentially expressed genes. Yellow bar, upregulated DEGs; blue bar, downregulated DEGs. Figure S3: List of enriched pathways in KEGG pathway enrichment analysis. Table S1: List of top upregulated (FC > 5) and top downregulated (FC < −10) genes after 4.0 μg/L thiram treatment. Table S2: List of primers used for qPCR. Supplementary File S1: Total list of DEGs obtained in high-dose (4.0 μg/L) thiram-treated zebrafish (HTZF) (FC > 2 and FC < −2). Video S1: video showing the heartbeats of DMSO-treated zebrafish embryos and 4.0 μg/L thiram-treated zebrafish embryos. Videos were captured under a stereomicroscope (Stemi 508, Zeiss, Germany) at 96 hpf.
