# Supplementary material for: Transcriptome Analysis of Thiram-Treated Zebrafish (Danio rerio) Embryos Reveals Disruption of Reproduction Signaling Pathways
Source: Biology (Basel). 2023 Jan 19;12(2):156. doi: 10.3390/biology12020156 (PMC9953208; doi:10.3390/biology12020156)
Supplement: Supplementary file 1 [file biology-12-00156-s001.zip › Supplimentory data.pdf]

Original research article

Transcriptome Analysis of Thiram-treated Zebrafish (*Danio rerio*) Embryos Reveals Disruption of Reproduction Signaling Pathways

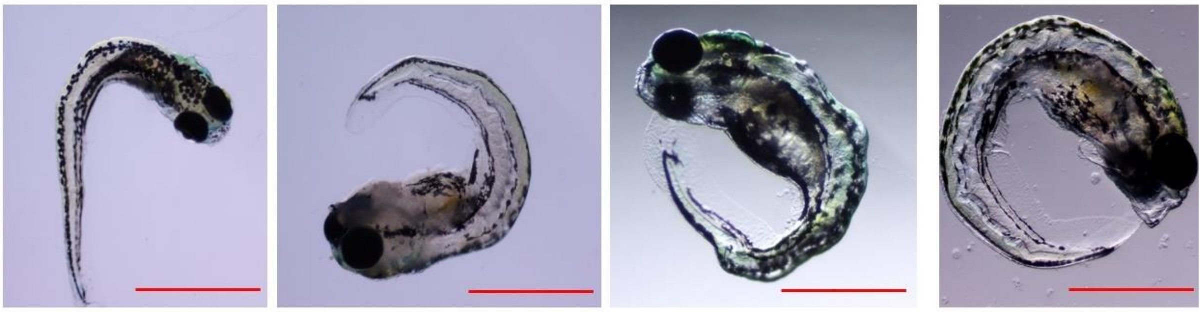

Figure S1. Representative images showing spinal curves observed in HTZF (zebrafish treated with 4.0 μg/L thiram); Scale = 0.5mm.

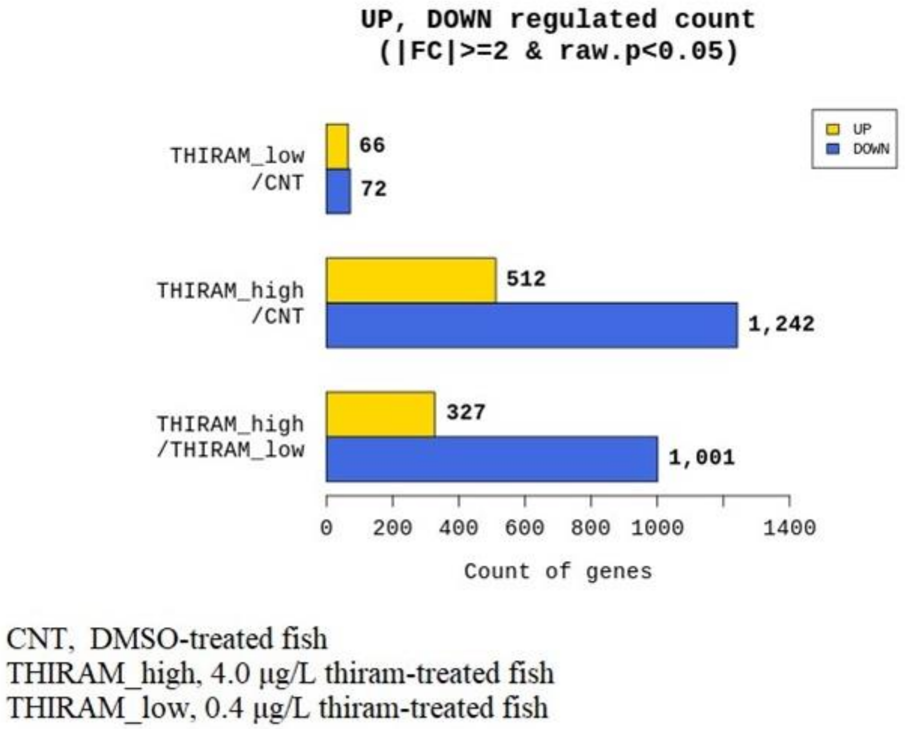

Figure S2. Up and down-regulated count of Difereentially Expresssed Genes. Yellow bar, upregulated DEGs; blue bar, downregulated DEGs.

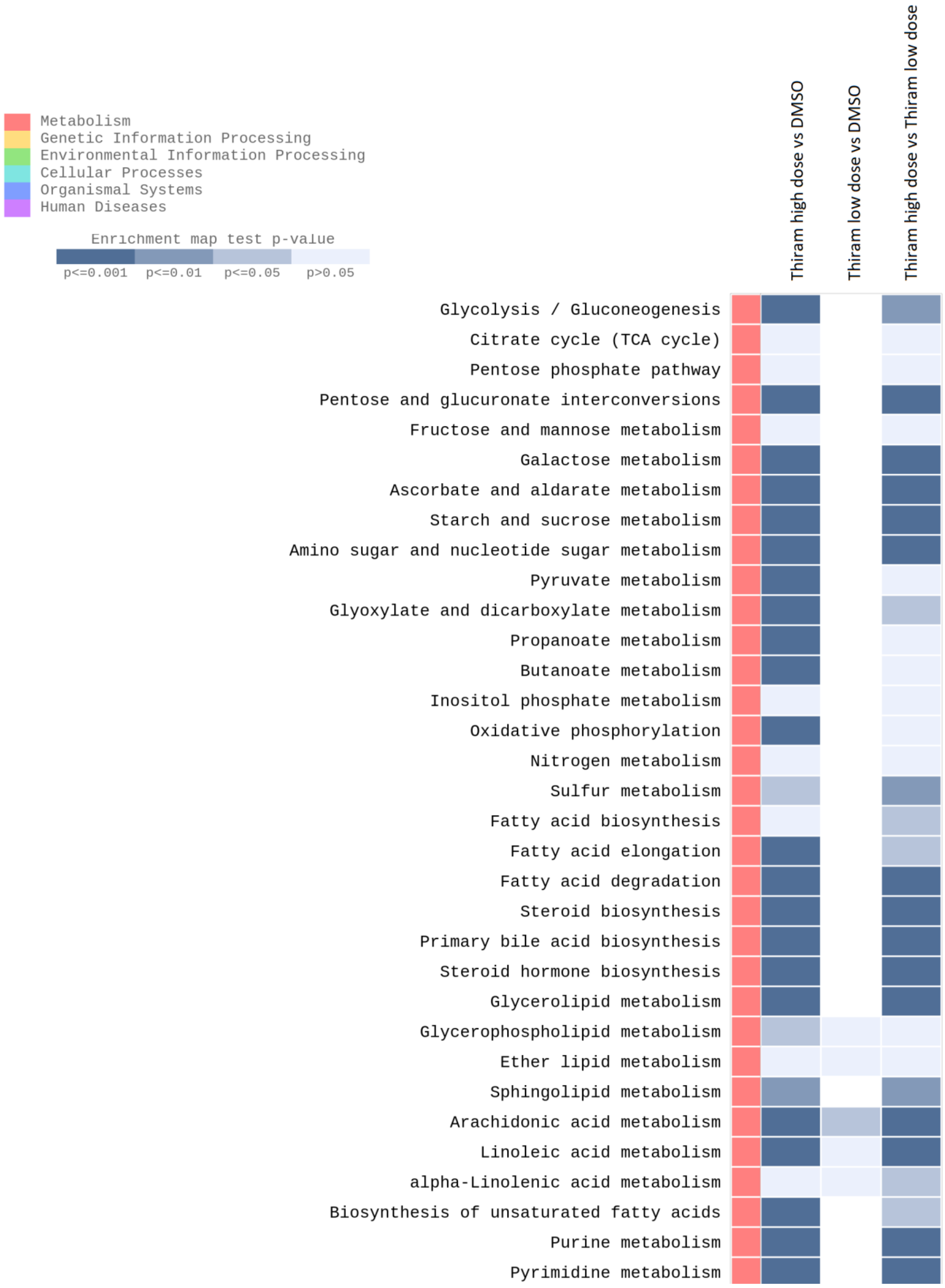

Metabolism
Genetic Information Processing
Environmental Information Processing
Cellular Processes
Organismal Systems
Human Diseases
Enrichment map test p-value
p<=0.001
p<=0.01
p<=0.05
p>0.05
Thiram high dose vs DMSO
Thiram low dose vs DMSO
Thiram high dose vs Thiram low dose
Glycolysis / Gluconeogenesis
Citrate cycle (TCA cycle)
Pentose phosphate pathway
Pentose and glucuronate interconversions
Fructose and mannose metabolism
Galactose metabolism
Ascorbate and aldarate metabolism
Starch and sucrose metabolism
Amino sugar and nucleotide sugar metabolism
Pyruvate metabolism
Glyoxylate and dicarboxylate metabolism
Propanoate metabolism
Butanoate metabolism
Inositol phosphate metabolism
Oxidative phosphorylation
Nitrogen metabolism
Sulfur metabolism
Fatty acid biosynthesis
Fatty acid elongation
Fatty acid degradation
Steroid biosynthesis
Primary bile acid biosynthesis
Steroid hormone biosynthesis
Glycerolipid metabolism
Glycerophospholipid metabolism
Ether lipid metabolism
Sphingolipid metabolism
Arachidonic acid metabolism
Linoleic acid metabolism
alpha-Linolenic acid metabolism
Biosynthesis of unsaturated fatty acids
Purine metabolism
Pyrimidine metabolism

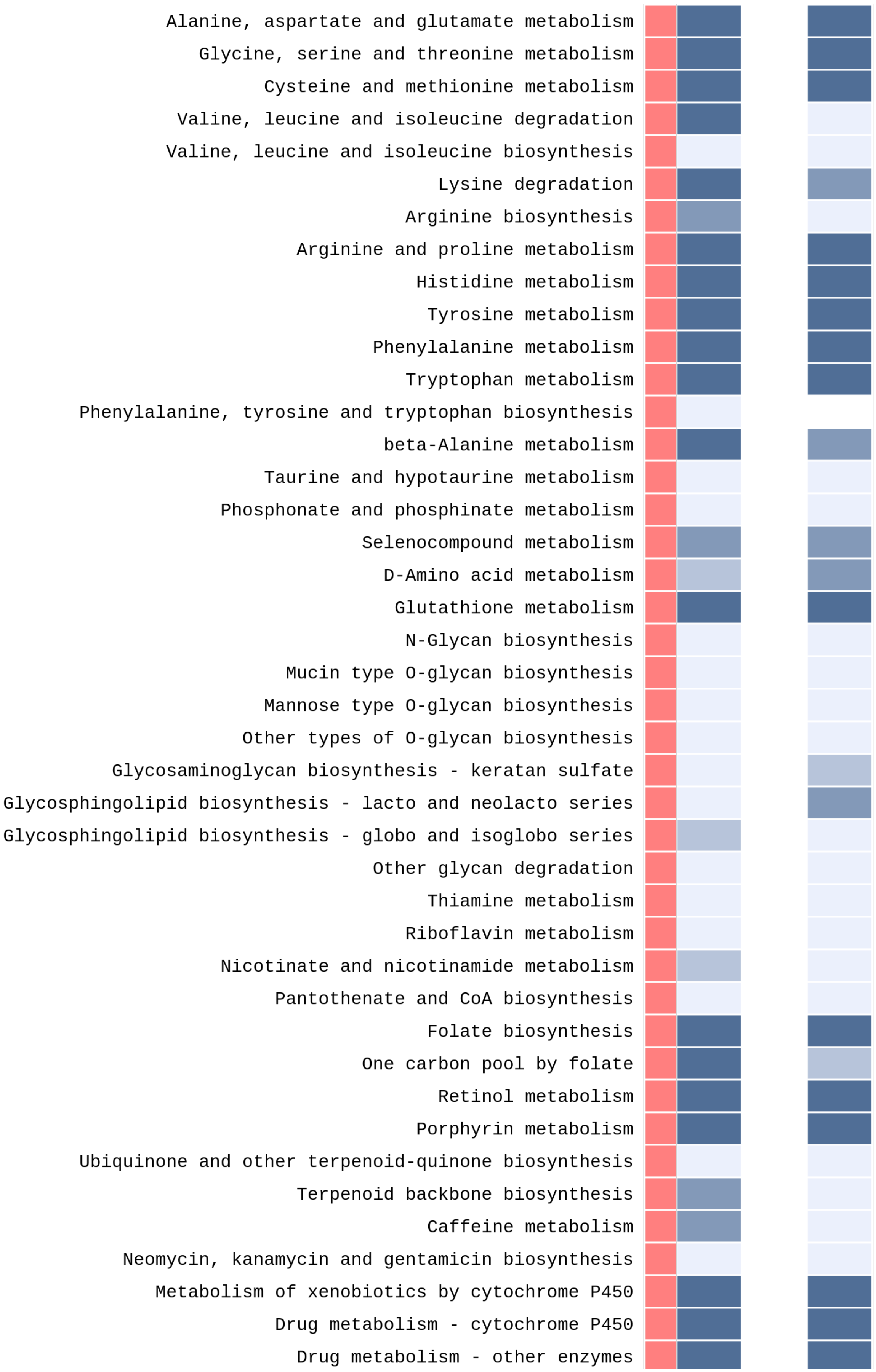
Alanine, aspartate and glutamate metabolism
Glycine, serine and threonine metabolism
Cysteine and methionine metabolism
Valine, leucine and isoleucine degradation
Valine, leucine and isoleucine biosynthesis
Lysine degradation
Arginine biosynthesis
Arginine and proline metabolism
Histidine metabolism
Tyrosine metabolism
Phenylalanine metabolism
Tryptophan metabolism
Phenylalanine, tyrosine and tryptophan biosynthesis
beta-Alanine metabolism
Taurine and hypotaurine metabolism
Phosphonate and phosphinate metabolism
Selenocompound metabolism
D-Amino acid metabolism
Glutathione metabolism
N-Glycan biosynthesis
Mucin type O-glycan biosynthesis
Mannose type O-glycan biosynthesis
Other types of O-glycan biosynthesis
Glycosaminoglycan biosynthesis - keratan sulfate
Glycosphingolipid biosynthesis - lacto and neolacto series
Glycosphingolipid biosynthesis - globo and isoglobo series
Other glycan degradation
Thiamine metabolism
Riboflavin metabolism
Nicotinate and nicotinamide metabolism
Pantothenate and CoA biosynthesis
Folate biosynthesis
One carbon pool by folate
Retinol metabolism
Porphyrin metabolism
Ubiquinone and other terpenoid-quinone biosynthesis
Terpenoid backbone biosynthesis
Caffeine metabolism
Neomycin, kanamycin and gentamicin biosynthesis
Metabolism of xenobiotics by cytochrome P450
Drug metabolism - cytochrome P450
Drug metabolism - other enzymes

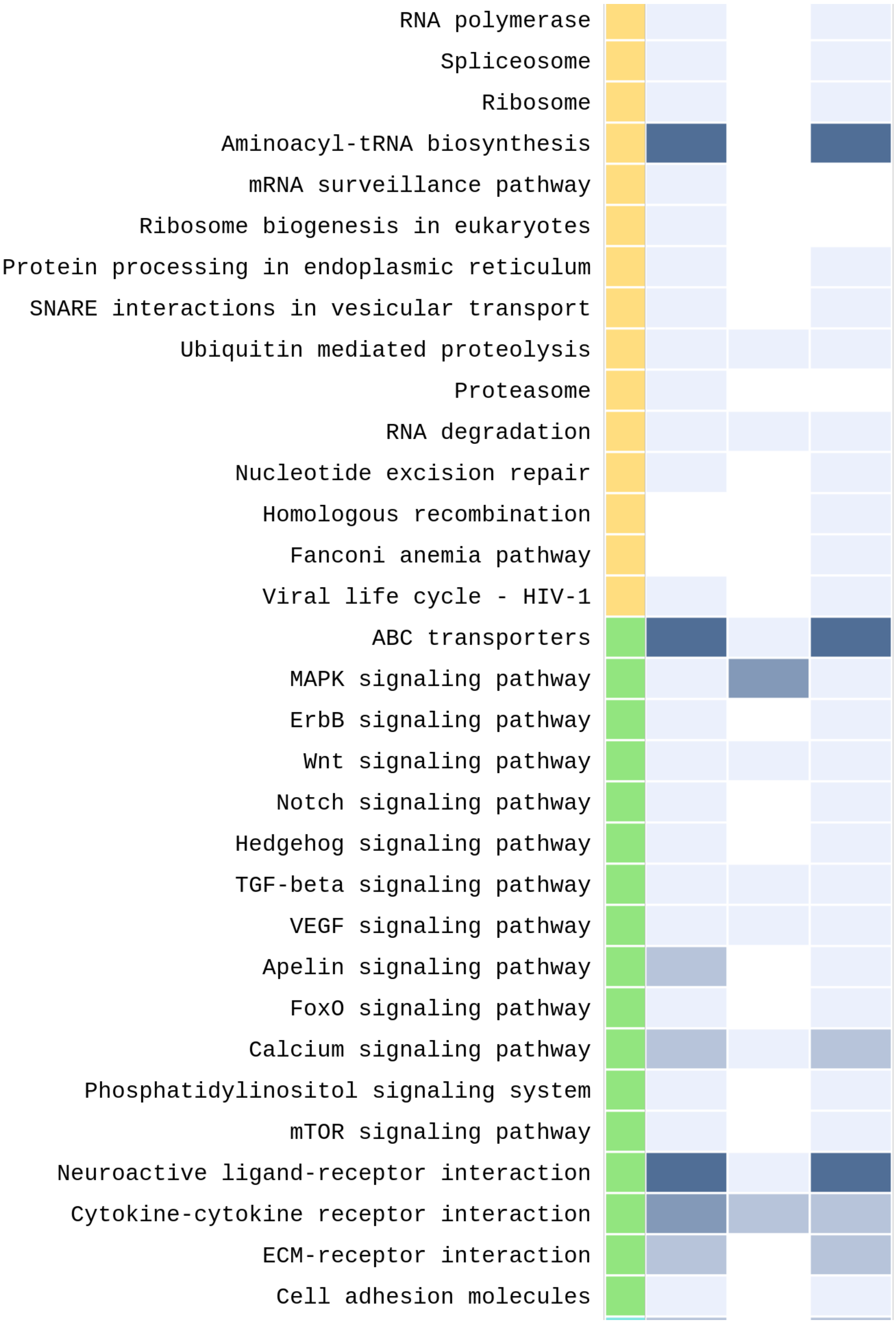
RNA polymerase
Spliceosome
Ribosome
Aminoacyl-tRNA biosynthesis
mRNA surveillance pathway
Ribosome biogenesis in eukaryotes
Protein processing in endoplasmic reticulum
SNARE interactions in vesicular transport
Ubiquitin mediated proteolysis
Proteasome
RNA degradation
Nucleotide excision repair
Homologous recombination
Fanconi anemia pathway
Viral life cycle - HIV-1
ABC transporters
MAPK signaling pathway
ErbB signaling pathway
Wnt signaling pathway
Notch signaling pathway
Hedgehog signaling pathway
TGF-beta signaling pathway
VEGF signaling pathway
Apelin signaling pathway
FoxO signaling pathway
Calcium signaling pathway
Phosphatidylinositol signaling system
mTOR signaling pathway
Neuroactive ligand-receptor interaction
Cytokine-cytokine receptor interaction
ECM-receptor interaction
Cell adhesion molecules

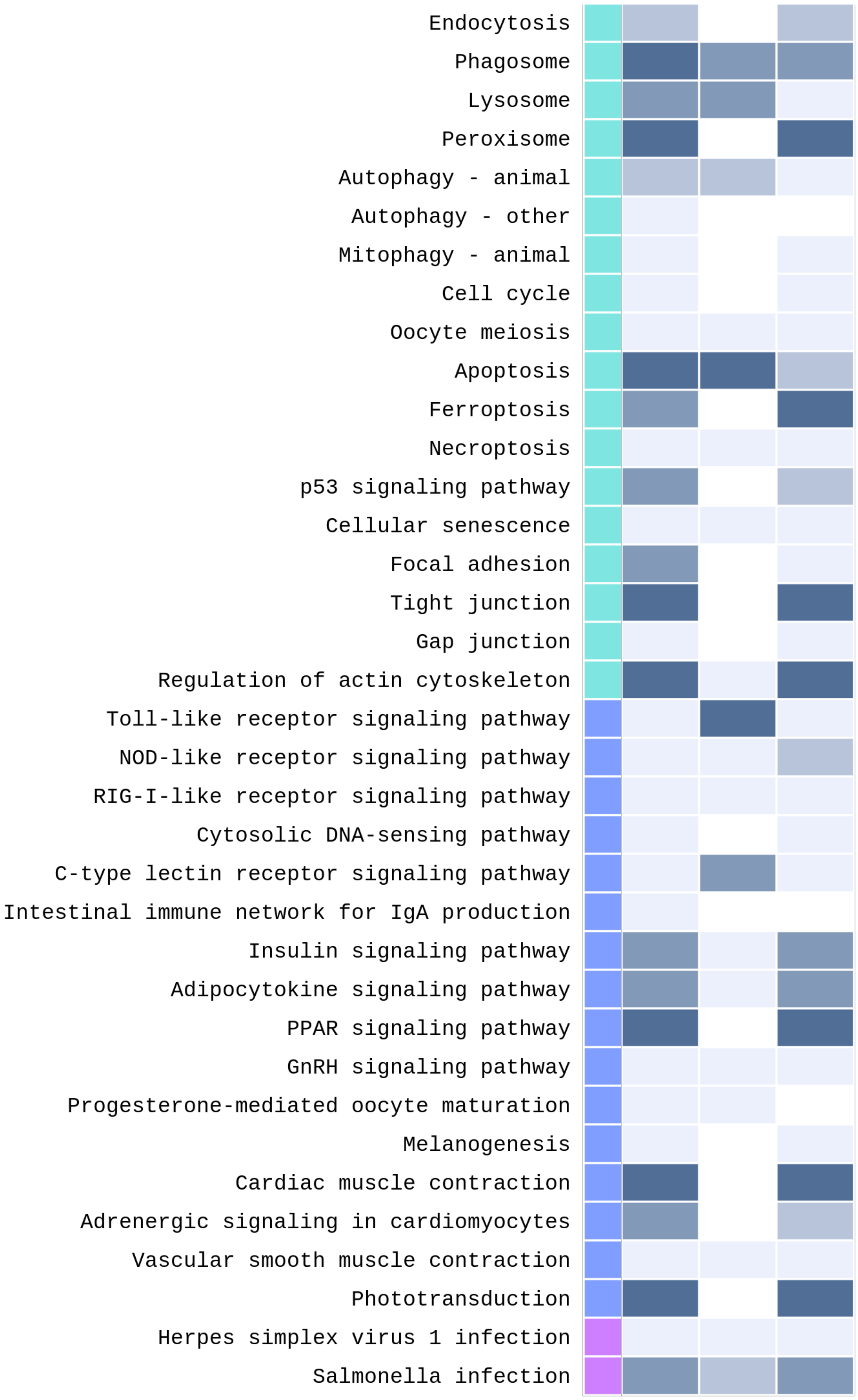

Figure S3. List of enriched pathway in KEGG pathway analysis

Supplementary Table 1. List of top upregulated (Fc > 5) and top downregulated (Fc < -10) gens after 4.0 μg/L thiram treatment

Transcript_ID	Gene_ Symbol	Description	THIRAM _high vs DMSO.fc
NM_001045282	zgc:136461	zgc:136461	-136.787033
NM_001024408,XM_005171867,XM_021476165	ela3l	elastase 3 like	-88.746940
NM_001139464	ela2	elastase 2	-57.165890
NR_015620	sycn.2	syncollin, tandem duplicate 2	-43.916148
NM_213249	chia.2	chitinase, acidic.2	-38.589898
NM_199886	ela2l	elastase 2 like	-37.621427
XM_021479024	LOC559107	lactase-phlorizin hydrolase	-36.644986
NM_001020482	cpa1	carboxypeptidase A1 (pancreatic)	-28.059968
NM_001077598	aoc1	amine oxidase, copper containing 1	-27.596219
NM_001076723	ugt5a2	UDP glucuronosyltransferase 5 family, polypeptide A2	-27.102873
XM_005162727,XM_009297410	si:ch211-161h7.5	si:ch211-161h7.5, transcript variant X2	-26.339568
NM_001177498	ugt5a4	UDP glucuronosyltransferase 5 family, polypeptide A4	-24.675914
XM_017352058	si:ch211-219a15.4	si:ch211-219a15.4	-21.130032
NM_001040175,XM_005164755	slc26a3.2	solute carrier family 26 (anion exchanger), member 3, tandem duplicate 2	-18.275764
XM_001921697,XM_021477806	LOC100147904	zonadhesin, transcript variant X1	-18.156202
NM_001099240	zgc:154142	zgc:154142	-17.294006
NM_001007356	cyp2n13	cytochrome P450, family 2, subfamily N, polypeptide 13	-17.191939
NM_001030281	mep1a.1	meprin A, alpha (PABA peptide hydrolase), tandem duplicate 1	-16.950245
XM_021470771	muc2.1	mucin 2.1	-16.764632
NM_001017724	zgc:112160	zgc:112160	-16.346380
NM_205643	pnp4b	purine nucleoside phosphorylase 4b	-16.264116

NM_001045385	Gck	glucokinase (hexokinase 4)	-16.146532
XM_017351817	si:ch211-214p16.3	si:ch211-214p16.3	-16.082961
NM_001089325	Anpepb	alanyl (membrane) aminopeptidase b	-16.059259
NM_001003729	zgc:92137	zgc:92137	-15.900719
XM_001343386	Eevs	2-epi-5-epi-valiolone synthase	-15.859847
XM_021468040	LOC110438125	zonadhesin-like	-15.817021
XM_009297092,XM_021470040	si:ch1073-138d10.2	si:ch1073-138d10.2, transcript variant X2	-15.301128
NM_001002076	fabp6	fatty acid binding protein 6, ileal (gastrotropin)	-14.773093
XM_021478559	LOC100331417	glycosyltransferase family 92 protein F13G3.3-like	-14.299863
NM_001007297,XM_005169359,XM_005169360	ace2	angiotensin I converting enzyme 2	-14.111774
NM_001109843	rhcgl1	Rh family, C glycoprotein, like 1	-13.463241
NM_131788,XM_005155405	cry5	cryptochrome circadian clock 5	-13.403722
NM_001033097	plac8.1	placenta-specific 8, tandem duplicate 1	-13.187097
NM_001102388	ankrd1b	ankyrin repeat domain 1b (cardiac muscle)	-13.039337
NM_199736	slc6a19b	solute carrier family 6 (neutral amino acid transporter), member 19b	-12.814183
XM_009293262,XM_021476927	vwa11	von Willebrand factor A domain containing 11, transcript variant X1	-12.745574
XM_003199562	si:ch1073-13h15.3	si:ch1073-13h15.3	-12.563861
NM_001002368,XM_005158777	ctsl.1	cathepsin L.1	-12.497900
XM_005156429	LOC101882496	sushi, nidogen and EGF-like domain-containing protein 1	-12.385683

XM_021466343	LOC110437898	uncharacterized LOC110437898	-12.273213
NM_001320118	dpep1	dipeptidase 1 (renal)	-12.225874
XM_021468039	wu:fb63a08	wu:fb63a08	-12.221217
NM_001025188	alpi.2	alkaline phosphatase, intestinal, tandem duplicate 2	-11.771664
NM_001159584	itln3	intelectin 3	-11.634496
rna-trnD	trnD	tRNA-Asp	-11.555799
NM_198064	slc15a1b	solute carrier family 15 (oligopeptide transporter), member 1b	-11.453348
NM_001098772	zgc:165502	zgc:165502	-11.362849
XM_003200878	si:dkey-22f5.9	si:dkey-22f5.9	-11.275452
rna-trnE	trnE	tRNA-Glu	-11.168690
NM_212618	ctrb1	chymotrypsinogen B1	-10.778630
XR_223934	si:dkey-104n9.1	si:dkey-104n9.1	-10.663062
NM_131624	slc34a2a	solute carrier family 34 (type II sodium/phosphate cotransporter), member 2a	-10.561936
NM_199605	prss59.1	protease, serine, 59, tandem duplicate 1	-10.274042
NM_001077456	zgc:153968	zgc:153968	-10.265950
NM_001002349	aqp10a	aquaporin 10a	-10.257251
NM_001083007,XM_021480002	zgc:153932	zgc:153932	-10.194839
NM_001081497,XM_005159300	slc28a1	solute carrier family 28 (concentrative nucleoside transporter), member 1	-10.131767
NM_001037438	cyp3a65	cytochrome P450, family 3, subfamily A, polypeptide 65	-10.105329
NM_183347	sult1st2	sulfotransferase family 1, cytosolic sulfotransferase 2	-10.041400
XM_021467200,XM_021467205,XM_021467206	myo7bb	myosin VIIBb, transcript variant X2	-10.020172
XM_021468701,XR_002456254	LOC110438198	uncharacterized LOC110438198, transcript variant X1	5.094741
NM_001037674	Smtlb	somatolactin beta	5.095720
XM_003198188,XM_021474762	sdr42e2	short chain dehydrogenase/reductase family 42E, member 2, transcript variant X2	5.129626

NM_001145614	ankrd37	ankyrin repeat domain 37	5.165035
XM_017351489	si:dkey-34k9.3	si:dkey-34k9.3	5.256805
NM_205569	Fosab	v-fos FBJ murine osteosarcoma viral oncogene homolog Ab	5.429473
NM_001327818	LOC557301	cocaine- and amphetamine-regulated transcript protein-like	5.447309
NM_001113589	hsp70l	heat shock cognate 70-kd protein, like	5.453033
XM_005164772	LOC101882092	podocan-like protein 1	5.472315
NM_001002493,XM_005160857	jdp2b	Jun dimerization protein 2b	5.731857
gene-LOC110438683	LOC110438683	.	6.012371
XM_682306,XR_658820	tgm2l	transglutaminase 2, like, transcript variant X1	6.096538
NM_213310,XM_005169814	egln3	egl-9 family hypoxia-inducible factor 3	6.141942
XR_002456031	LOC110438090	uncharacterized LOC110438090	6.472505
XM_017352961,XM_021469064,XR_002456325	LOC798694	complement component C6-like, transcript variant X1	6.477369
XR_002459390,XR_002459391,XR_002459392,XR_002459393,XR_002459394	im:7152855	im:7152855, transcript variant X1	6.669534
XR_002458203,XR_002458204	LOC110439372	uncharacterized LOC110439372, transcript variant X1	6.796486
XM_001919491	si:ch73-265d7.2	si:ch73-265d7.2	6.819292
XR_002455919	LOC110438028	uncharacterized LOC110438028	6.836930
XM_021478389,XM_021478390	si:dkey-21p1.3	si:dkey-21p1.3, transcript variant X2	7.556664
NM_001305571	Tcap	titin-cap (telethonin)	7.685431
NM_199950	socs3a	suppressor of cytokine signaling 3a	8.215998
NM_001033740,XM_005165183	dao.1	D-amino-acid oxidase, tandem duplicate 1	11.262813
NM_001326701	hbae5	hemoglobin, alpha embryonic 5	15.261024

Supplementary Table 2. List of oligo primers used in qPCR validation

**Gene (Sequence ID)**	**Orientation**	**Oligo sequence**
hbae5 (NM_001326701)	Forward	TGAATTGCACGCCTTTCAGC
	Reverse	GCGGTGAAGTCATCAGGGAA
socs3a (NM_199950)	Forward	ATGGAGTTGTCCAGCCACTG
	Reverse	GCAAGAATGGCGCTTCAACA
tcap (NM_001305571)	Forward	TGACTGGCGCAGCATCAATA
	Reverse	GAGGACGCACCTGCCAATAA
egln3 (NM_213310)	Forward	TTTGGCTTTTGCGCAGGTTG
	Reverse	GGAATGCATGCGTTTGACCT
jdp2b (NM_001002493)	Forward	CTGGCGGAACTTTACCGTCT
	Reverse	CAAACGGTCTTTTGGCGTGT

smtlb (NM_001037674)	Forward	CTGCTTCCGAAAAGACGCAC
	Reverse	GGGAGCAGTTTTCCCTGTCA
ela2 (NM_001139464)	Forward	TGATCGACAAGCAGTGGGTC
	Reverse	AGGTTGTGCTTGCCCAGTAA
chia.2 (NM_213249)	Forward	TGGATAATTGCGGAGCAGGC
	Reverse	AATGTGCAGTGTCCCATTTCA
aoc1 (NM_001077598)	Forward	GACTCCAGGAAACTCGGTGG
	Reverse	TGACCCTTTTCATCGGGACG
ugt5a4 (NM_001177498)	Forward	TTTGATGCTGACTTCGGGGA
	Reverse	CAGCAAGTGCCATTTCAGTCC
β-actin (Housekeeping gene)	Forward	CGAGCTGTCTTCCCATCCA
	Reverse	TCACCAACGTAGCTGTCTTTCTG
